# Evaluation of a flexible assertive community treatment (FACT) program for patients with severe mental illness: an observational study in Salzburg, Austria

**DOI:** 10.1186/s13033-024-00628-8

**Published:** 2024-02-09

**Authors:** Matthias Gerhard Tholen, Anna Martin, Theresa Stemeseder, Thomas Vikoler, Barbara Wageneder, Wolfgang Aichhorn, Andreas Kurt Kaiser

**Affiliations:** 1 Psychotherapy and Psychosomatics, University Hospital of Psychiatry, Paracelsus Medical University, Salzburg, Austria; 2 Department of Psychology, University of Salzburg, Salzburg, Austria

**Keywords:** Assertive community treatment, Flexible assertive community treatment, Mental health care, Severe mental illness

## Abstract

**Background:**

Inpatient treatment of severe mentally ill patients binds substantial resources and creates the dilemma of “revolving-door hospitalizations”. Evidence suggests that these patients benefit more from an assertive outreach community psychiatric treatment. This descriptive study evaluates the implementation of a new treatment program for severe mentally ill patients provided by a flexible assertive community treatment (FACT) team.

**Methods:**

An uncontrolled design with routine data was used to measure the total length of stays, readmission rates and number of contacts one year prior to the implementation of the FACT program and the following first three years of treatment.

**Results:**

A continuous decrease of hospitalization among patients with severe mental illness was observed with the implementation of the FACT program with declines in total length of stays and readmission rates and accompanied with a decreasing number of contacts per year.

**Conclusion:**

Our findings indicate that this program may create effects in stabilizing patients with severe mental illness and may be highly relevant also for other patient groups.

## Background

Severe mental illnesses (SMI) are characterized by chronic mental, affective and social impairments with typically long histories of treatment—intra- and extramural. Patients with SMI generally present diagnoses from schizophrenia-spectrum disorders [1, 2], bipolar disorders, or severe affective disorders, like major depression with psychotic features or severe anxiety [2]. A central feature of SMI constitutes impairments in everyday functioning [3], including deficits in social and occupational functioning, residential maintenance, medication management, and basic self-care [4–8]. Consequentially, patients with SMI show a lower life expectancy of 10–20 years compared to the general population and a higher probability to die of an unnatural cause such as an accident or a suicide [9].

Professional treatment faces challenges in maintaining the compliance of patients with SMI [10–14]. Multiple studies demonstrate a relation between a lower socioeconomic status, lower educational level, psychoeducative lack [12–14], and low family support [15] with non-compliance in treatment among patients. Poor social functioning of patients with SMI may lead to discontinuation of extramural long-term treatments [16, 17] resulting in frequent hospitalizations, in most cases in form of acute admissions [4].

The assertive community treatment (ACT) implemented by Test and Stein [18, 19] is considered to be one of the leading models of community psychiatric health care systems developed for patients with SMI [20]. ACT provides outreach community psychiatric treatment and care to prevent hospital stays, improve treatment adherence and rehabilitation [21]. The program promotes de-institutionalization and reintegration into society [4] by means of comprehensive, individualized and community-oriented care. Core of the ACT are the multidisciplinary teams and a low patient-staff ratio which enables regular contacts [2, 5]. Practitioners have a precise insight into the patient’s living environment providing an intensive and targeted treatment to support the patients’ needs, e.g., regular medication intake. If possible, ACT teams work with family members giving psychoeducation and support and involving them in the treatment plan. Further key principles are integration of services, team approach, locus of contact in the community, focus on everyday problems in living, rapid access, assertive outreach, individualized services and time-unlimited services [2].

In several evaluation studies ACT programs showed overall positive effects: a reduction of admissions and stays in hospitals, readmission rates and involuntary admissions [2, 4, 5, 22–29], improvements in symptoms, quality of life, and level of functioning [2, 23, 26, 28–30]. Patients were more likely to engage in treatment and to stay adherent to medication [2, 23, 24]. They and their relatives were more satisfied with the ACT health care system [23, 24, 28, 31] compared to standard health care. A meta-analysis of Vanderlip and colleagues [29] showed that, despite the additional costs for the implementation and maintenance of ACT teams, there was no increase in overall costs due to the reduction in hospitalizations.

Van Veldhuizen and Bähler [32] adapted the ACT model with the aim of combining recovery-oriented care, evidence-based medicine and best practices, integrated community and hospital care. Flexible ACT (FACT) is a further development of the ACT model (other developments comprise e.g., therapeutic ACT [33–35], or resource-group ACT [36]). Standard ACT focuses on crisis intervention and stabilization. It does not target all patients with SMI, but rather focuses on the most severe cases—primarily unstable, psychotic patients who require frequent readmissions. Many of them have personality disorders, substance abuse issues, low medication compliance, and a tendency to avoid treatment. It is estimated that this group of severe cases constitutes approximately 20% of the entire group of long-term psychiatric patients [2]. While ACT teams provide treatment involving active outreach and follow-up care, FACT aims to cover the entire group of people with SMI, which includes both the 20% group of severe cases and the other 80%. As FACT teams provide individual case management and need-based treatment with a focus on recovery and empowerment, a larger number of patients can be included. Another advantage is that patients can be resumed after a drop-out directly without a waiting list.

Since the FACT model was implemented in the Netherlands [32, 37, 38], it has spread to a large number of health care teams in Belgium [39], Sweden [40], Norway [41], England [42, 43], Denmark [44] and Canada [45]. A recent evaluation of the FACT model demonstrates an increase of collaborations between different service providers within a complex mental health system [46]. Despite its growing importance within different multinational mental health care systems, this treatment form requires further scientific evidence in terms of its efficacy [44, 47]. This observational study aims to evaluate the efficacy of treatment of patients with SMI within the first implementation of FACT in Austria on the basis of the total length of stay, readmissions, and the number of outpatient contacts.

## Methods

### Study sample and design

A descriptive study was designed at the University Hospital of Psychiatry, Psychotherapy and Psychosomatics, Salzburg, Austria to evaluate the implementation of a treatment programm provided by a FACT team. The study was approved by the local ethics committee (reference number 1020/2019). Participation in the evaluation was voluntary. All participants gave written informed consent to partake in the evaluation of the FACT program. This study covers an observational period of four years, one year prior to the admission into the FACT program and the following first three years during treatment.

The FACT program was delivered in the city of Salzburg and the surrounding northern part of Salzburg province with 351 013 inhabitants (2011 census). Patients were exclusively recruited during the course of an inpatient stay. In case of suspected revolving door hospitalization (based on the medical history), the senior psychiatrist provides a referral to the FACT team after medical examination and diagnosis. A team member conducts an interview with the patient regarding the patient’s needs and goals and the inclusion criteria of the FACT program. The inclusion criteria for patients encompass the presence of a SMI indicated by (i) diagnosis (schizophrenic, bipolar or severe affective disorder), (ii) age > 18, (iii) prolonged (≥ 28 days) or frequent hospitalizations (≥ 2) during the last twelve months, (iv) severe functional impairment (e.g., inability to engage social relationships, or to keep a job), and (v) a high need for continuous support (e.g., inability to participate in life without external help). If the patient meets the necessary requirements, the case is then reviewed by the whole FACT team to allocate responsibilities (e.g. case management) and the patient is introduced to other members of the team with different professions (psychiatrist, clinical psychologist, social workers, psychiatric nurses).

**Fig. 1:**
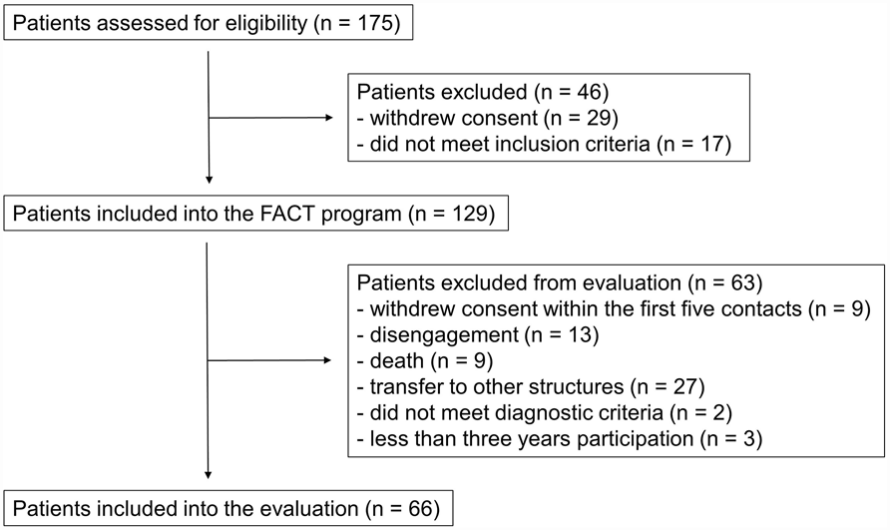
A CONSORT diagram shows the documentation of the absolute numbers of participants in the evaluation of the FACT program

Between March 2018 and March 2021, a total of 175 patients were assessed for eligibility (Fig. 1). Forty-six patients were not included into the FACT program due to patient’s refusal (29) or the patients did not meet inclusion criteria (17). Out of 129 patients that were recruited for the FACT program 66 patients (51.2%) were treated for at least three years. Nine patients (7.0%) withdrawed their decision to take part in the FACT program within the first five contacts. During the three years observation period nine patients died (7.0%), 13 patients (10.1%) disengaged from service, and 27 patients (20.9%) were transferred to other structures (e.g., assisted living). Another three patients (2.3%) that were treated less than three years and two patients (1.6%) that did not meet the diagnostic criteria were excluded from the evaluation.

### Intervention

The FACT program includes an individual case management as well as an assertive outreach care. The team, consisting of a psychiatrist, a clinical psychologist, social workers and psychiatric nurses support patients in their rehabilitation and recovery processes. All patients have an individual case manager, who visits the patient at home unless a patient prefers to attend appointments in the clinic as social skills training. Regular contacts take place once a week, with increasing stabilization every 2 weeks or in longer intervals. The frequency of contacts can flexibly adapt according to the needs of the patients. The case manager accompanies patients to social service agencies, medical appointments, and supports patients in terms of medication setting and management, inclusion in the family and community, or stabilization of financial security according to the treatment plan which is based on the patient’s needs and personal goals. During the regular contacts, the case manager carefully monitors signs of destabilization to prevent crises or acute hospitalizations. If necessary, the patients receive an intensive short-term care from all professionals of the FACT team (shared caseload) on a daily basis or a short-term hospital admission is arranged to stabilize the patient. All patients admitted to a hospital are visited weekly preparing their discharge and support at home. In case of a long-term stabilization the patient may decide to terminate the FACT program with the option to re-enter in case of worsening of symptoms.

### Measures

Clinical routine data were extracted from an automated hospital information system and were analyzed by the R software 4.2.3 (2023‑03‑16) and R Studio 2023.03.0 + 386. Descriptive statistics, density plots and a Kaplan Meier plot were used to demonstrate the results for the number of contacts, total length of stay and the readmission rates for the totalized inpatient and day clinic stays as an indicator of efficacy of the FACT program. Non-parametric Friedman tests of differences among repeated measures were conducted. In case of significance pair-wise Wilcoxon signed rank tests were performed at a statistical threshold of *p* < 0.05.

## Results

### Participant characteristics

The modus of gender was female (*n* = 45, 68%). Participants’ age at the start of the program ranged between 21.6 and 78.9 years (mean = 50.8 years, *sd* = 14.7). They were generally diagnosed with schizophrenia or another psychotic disorder (*n* = 39, 59.1%), bipolar disorder (*n* = 11, 16.7%), or severe major depressive disorder (*n* = 16, 24.2%). The presence of one or more comorbid psychiatric diagnoses (*n* = 40, 60.6%) was prevalent and comprised substance use disorder (*n* = 22, 33.3%), posttraumatic stress disorder (*n* = 11, 16.7%), anxiety disorder (*n* = 9, 13.6%), obsessive compulsory disorder (*n* = 4, 6.1%), and other (e.g. major depressive disorder (*n* = 4, 6.1%), organic disorder (*n* = 4, 6.1%), or personality disorder (n = 5, 7.6%)).

### Number of contacts per year

The number of contacts in the psychiatric outpatient clinic are significantly declining during the course of four years (χ^2^(3) = 18.82, *p* < 0.001). Pairwise comparisons reveal a suggestive difference between pretreatment and the second year (*p* = 0.09) and a significant difference between pretreatment and the third year (*p* = 0.03) of the FACT program (Table 1). At the same time, the number of contacts within the FACT program show a continuous significant decrease (χ^2^(2) = 63.79, *p* < 0.001). Post hoc comparisons show that the frequency of regular contacts decreases significantly from more than once a week to less than once a week in one year (*p* < 0.001) and to once every ten days (*p* < 0.001) in the subsequent year.

**Table 1:** Descriptive statistics

	Pretreatment	1st year	2nd year	3rd year
Number of contacts per year				
Psychiatric outpatient clinic, mean (SD)	1.6 (3.0)	1.5 (5.1)	1.2 (5.8)	1.3 (5.7)
FACT program, mean (SD)	–	56.8 (29.0)	43.6 (23.8)	35.8 (20.0)
Length of stay in days				
Mean (SD)	72.1 (53.7)	26.4 (44.4)	15.4 (29.2)	12.9 (23.9)
Range	13–225	0–198	0–141	0–111
Readmission rates per year				
Mean (SD) number of readmissions	1.4 (0.9)	0.6 (1.0)	0.5 (1.1)	0.3 (0.6)
Mean (SD) number of involuntary admissions	0.5 (0.8)	0.05 (0.2)	0.03 (0.2)	0 (0)
Total readmissions, number of patients (%)	–	22 (33.3)	14 (21.2)	18 (27.3)

Descriptive Statistics for the patients included into the evaluation (*n* = 66)

SD = standard deviation

### Length of stay

**Fig. 2:**
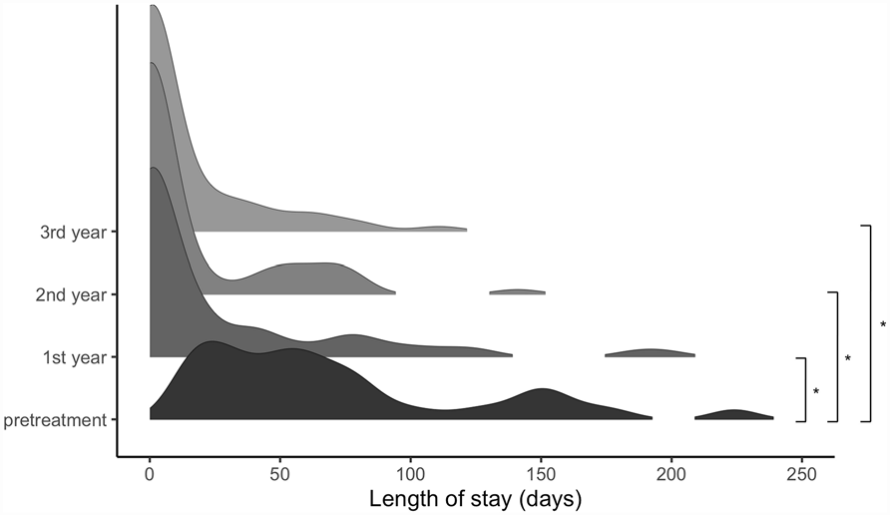
Density plots show the distribution of length of stays one year prior to the implementation (pretreatment) and the first three years after the implementation of the FACT program (n = 66)

The multimodal distribution of the pretreatment year changes into a highly right skewed unimodal distribution over the first three years of the FACT program (Fig. 2). Prior to the implementation of the program, patients stay in the hospital with a median length of stay of 55.5 days (30; 91.25). The middle 50% of the patients are hospitalized between one to three months. With the implementation of the FACT program the median length of stay fell down to zero with a shrinking interquartile range over the three years from zero to 37.5 days in the first year and from zero to 12.75 days in the second and third year. The length of stay reduces significantly (χ^2^(2) = 77.33, *p* < 0.001). Pairwise comparisons show a significant difference between pretreatment and the subsequent years within the FACT program (each *p* < 0.001). No significant differences are found between the first, second, and third year of treatment. Decreasing means and standard deviations indicate on the one hand a shift to dehospitalization and on the other hand a reduction of the extreme values, also reflected by a continual decrease of the total range over the three years (Table 1).

### Readmission rates

**Fig. 3:**
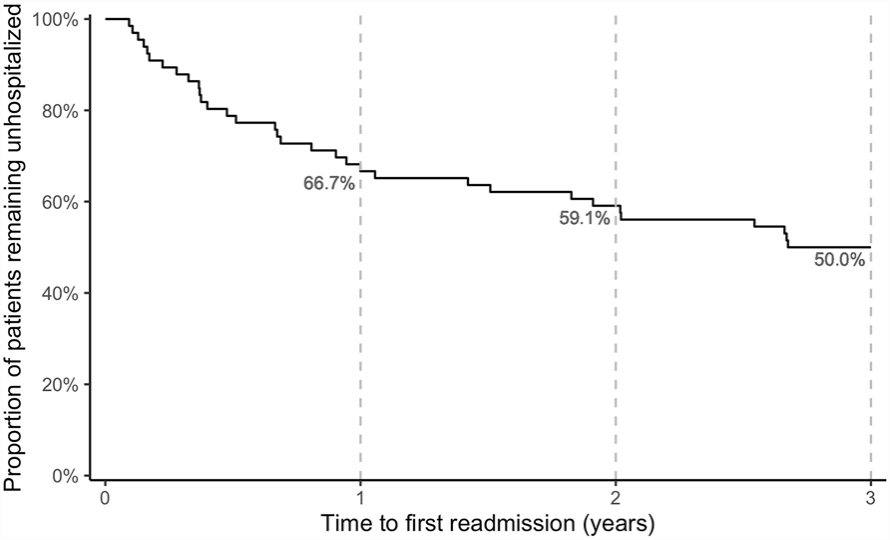
Kaplan Meier plot shows the time to first readmission during three year observation period

After three years, 50% of patients still remain unhospitalized (Fig. 3). Thirty-three patients were readmitted to hospital ranging between 34 and 977 days to first readmission. Most of them (*n* = 22, 33%) were readmitted during the first year with a decreasing proportion of readmissions over the second (*n* = 5, 7.6%) and third (*n* = 6, 9.1%) year. Relating to the number of patients that were readmitted, there is no clear trend in evidence (Table 1). The number of readmissions reduces significantly over the time (χ^2^(2) = 73.10, *p* < 0.001). Multiple comparisons reveal significant differences between pretreatment and the following three years within the FACT program (each *p* < 0.001). No differences are found between the first, second, and third year of treatment. The number of involuntary admissions due to self- or other-endangerment does also decrease significantly (χ^2^(2) = 51.48, *p* < 0.001). There are suggestive differences between pretreatment and the first (*p* = 0.077) and second year (*p* = 0.051) and a significant difference between pretreatment and the third year of the FACT program (*p* = 0.024) with no differences between the first, second, or third year of treatment.

## Discussion

Psychiatric hospitals are regularly monitored in terms of performance indicators, such as length of stay or readmission rates. This study aimed to evaluate the number of contacts, the total length of stay as well as readmission rates as an indicator of efficacy of the reorganization of the mental health care system for severe mentally ill patients along the lines of the FACT program. With the implementation of the FACT program we observed a continuous decrease of hospitalization and involuntary admissions among patients with SMI. Over the whole time of observation half of the patients remain unhospitalized. Even though the total readmissions do not show a clear trend and vary between 21 and 33%, readmitted patients show less frequent hospitalizations and shorter length of stays indicating that even those patients with the most severe forms of mental illnesses and the highest use of mental health care benefit from the FACT. Decreasing mean number of contacts per year may indicate a stabilization of the patients throughout the three years of treatment.

These results confirm other observational studies that reported dehospitalization effects in terms of fewer length of stays, admissions rates or outpatient contacts [37, 42, 43, 48]. Additionally, a recent quasi-experimental study found the FACT program to be associated with a higher number of contacts and fewer admissions compared to community mental health care or ACT [44]. Concurrent with these previous uncontrolled studies and the quasi-experimental controlled study, this evaluation is methodologically inadequate to demonstrate causation. Since this observational study focuses on the long-term effects of the treatment, a waiting list control group is not preferred for ethical reasons. A wait may be detrimental for patients with SMI resulting in worsening of symptoms. An alternative technique to construct an artificial control group (relating to the time period of investigation) via prospensity score matching [49, 50] cannot be applied as the treated patients are not comparable to other psychiatric patients due to differences concerning the selection criteria of the FACT program (e.g. severity of the psychiatric disorder, or functional impairment). All eligible patients with SMI were invited to participate in the program, following that patients from an articificial control group necessarily do not fulfil the selection criteria (selection bias). The creation of a historical control group based on routine data of patients that had been at the clinic ahead of the recruitment phase was not possible as patient’s data on functional impairment (inclusion criterion iv) and the need for continuous support (inclusion criterion v) were not systematically collected within the automated hospital information system.

Due to the lack of a control group, one may argue that the described results may be explained by a time effect. Given that patients with SMI are characterized by frequent or prolonged hospitalizations including involuntary admissions over a long period of time without substantial changes of functionality, we argue that the improvements are more likely associated with the implementation of the FACT program. The patient’s total length of stay, readmission rates as well as the number of contacts significantly decrease in the first year of the treatment and stabilize over the following years. The effects may be partially explained by a change of admission behaviour that may be associated with the implementation of the new treatment programm. The FACT team has a gatekeeper function for all treatments including inpatient or day clinic stays. For example, in the case of inpatient treatment, the team remains in contact with the patient and retains the role of coordinator for the overall treatment [51].

In order to endorse standard mental health care or FACT, further randomized controlled studies are required comparing the systems in terms of efficacy but this was beyond the scope of our evaluation. This observational study was limited to clinical data from the hospital information system and did not include information about level of functioning, adherence, client satisfaction or engagement in service, which are known positive outcomes in ACT [23]. Further studies are required to evaluate how patients social functioning and symptom severity is affected by the FACT program by using standardized measurements like global assessment of functioning (GAF) or clinical global impression (CGI).

New models in mental health care such as the recent developments of the ACT model like FACT may serve as qualification and innovation of the mental health system to accommodate existing structural deficiencies. With the intent to relieve the psychiatric inpatient care nowadays, the time spend in the hospital is generally being minimized with the result of higher frequencies of readmissions (“revolving-door hospitalization”). This practice may therefore not have an impact on the total length of stay of patients with SMI. Our results indicate that the total length of stay and the frequencies of readmissions reduce with the implementation of the FACT program. The FACT model may offer an opportunity to create sustainable effects in stabilizing the patients and reducing hospitalization rates. The positive outcomes of the FACT program may not only be ascribed to the direct interventions between patient and FACT team member but also to the network activity forming a bridge between primary care and specialist health services [46]. An important approach that may be highly relevant also for other patient groups, e.g., forensic patients [52], dementia patients [53], or adolescents with complex care needs [54].

## Conclusion

The implementation of a FACT program for patients with SMI is associated with a reduction of length of stays and readmission rates in psychiatry. These results may suggest a stabilizing effect. Further scientific evidence is required demonstrating beneficial effects of the FACT program for patients with SMI and other patient groups with high need for continuos support.

## Data Availability

The data of this study are available from the authors upon reasonable request.
